# Comparison of Ocular Ultrasonography and Magnetic Resonance Imaging for Detection of Increased Intracranial Pressure

**DOI:** 10.3389/fneur.2018.00278

**Published:** 2018-04-24

**Authors:** David F. Patterson, Mai-Lan Ho, Jacqueline A. Leavitt, Nathan J. Smischney, Sara E. Hocker, Eelco F. Wijdicks, David O. Hodge, John Jing-Wei Chen

**Affiliations:** ^1^Department of Ophthalmology, Mayo Clinic, Rochester, NY, United States; ^2^Department of Radiology, Mayo Clinic, Rochester, NY, United States; ^3^Department of Anesthesiology, Mayo Clinic, Rochester, NY, United States; ^4^Department Neurology, Mayo Clinic, Rochester, NY, United States; ^5^Department Health Sciences Research, Biomedical Statistics and Informatics, Mayo Clinic, Jacksonville, FL, United States

**Keywords:** optic nerve sheath, ultrasonography, idiopathic intracranial hypertension, magnetic resonance imaging, empty sella, pseudotumor cerebri

## Abstract

**Background/aims:**

To evaluate and compare the performance of ocular ultrasonography (US) and magnetic resonance imaging (MRI) for detecting increased intracranial pressure (ICP) in patients with idiopathic intracranial hypertension (IIH).

**Methods:**

Twenty-two patients with papilledema from IIH and 22 with pseudopapilledema were prospectively recruited based on funduscopic and clinical findings. Measurements of optic nerve sheath diameters (ONSDs) 3 mm behind the inner sclera were performed on B-scan US and axial T2-weighted MRI examinations. Pituitary-to-sella height ratio (pit/sella) was also calculated from sagittal T1-weighted MRI images. Lumbar puncture was performed in all patients with IIH and in five patients with pseudopapilledema.

**Results:**

Average US and MRI ONSD were 4.4 (SD ± 0.7) and 5.2 ± 1.4 mm for the pseudopapilledema group and 5.2 ± 0.6 and 7.2 ± 1.6 mm for the papilledema group (*p* < 0.001). Average MRI pit/sella ratio was 0.7 ± 0.3 for the pseudopapilledema group and 0.3 ± 0.2 for the papilledema group (*p* < 0.001). Based on receiver-operator curve analysis, the optimal thresholds for detecting papilledema are US ONSD > 4.8 mm, MRI ONSD > 6.0 mm, and MRI pit/sella < 0.5. Combining a dilated US ONSD or MRI ONSD with a below-threshold MRI pit/sella ratio yielded a sensitivity of 73% and specificity of 96% for detecting IIH. Adding the US ONSD to the MRI ONSD and pit/sella ratio only increased the sensitivity by 5% and did not change specificity.

**Conclusion:**

US and MRI provide measurements of ONSD that are well-correlated and sensitive markers for increased ICP. The combination of the ONSD and the pit/sella ratio can increase specificity for the diagnosis of IIH.

## Introduction

Idiopathic intracranial hypertension (IIH), also known as pseudotumor cerebri, is a disease characterized by increased intracranial pressure (ICP) without a mass lesion obstructing the ventricular system or other cause of raised ICP. Patients can develop significant papilledema and subsequent visual loss because of the effects of the increased ICP on the optic nerve. Patients can also rarely have IIH causing headaches without papilledema (1). Neuroimaging and lumbar puncture are necessary in confirming the diagnosis of IIH, as there are many other etiologies of increased ICP. The invasive nature of lumbar puncture comes with potential side effects including infection, bleeding, and over-drainage or leakage of cerebrospinal fluid (CSF) with resultant intracranial hypotension and headache.

In addition, there is variability in lumbar puncture opening pressures based on patient positioning and natural physiologic variation (2, 3). Valsalva maneuver alone can increase the opening pressure on lumbar puncture up to 47 cm H_2_O (4). CSF pressure measurements also may vary between standard lateral decubitus positioning and prone positioning with varying degrees of table tilt (5). Direct ICP monitoring with epidural, subdural, or preferably intraventricular devices provides more accurate measurements (2, 3) but is highly invasive and requires inpatient hospitalization. Therefore, non-invasive surrogate markers for detecting raised ICP are highly useful clinical tools in the evaluation of suspected increased ICP.

Many studies, most of which originate in the critical care literature, have investigated ultrasonography (US) measurements of the optic nerve sheath diameter (ONSD) as a marker of increased ICP (6–23). The advantages of US include its speed, reduced cost, non-invasive modality, relative ease of use, repeatability, and lack of side effects. Magnetic resonance imaging (MRI) has also been used to evaluate for indirect signs of increased ICP using various imaging signs including empty sella, dilation and tortuosity of optic nerve sheaths, effacement of subarachnoid space, slit ventricles, transverse venous sinus stenosis, and flattening of the posterior globes (14, 18, 24–31).

The goal of this study was to compare the performance of B-scan US and MRI in identifying increased ICP in patients with IIH *via* measurement of the ONSD and *via* calculation of the MRI-derived, pituitary-to-sella turcica (pit/sella) ratio to quantify the “empty sella” sign that can be seen with IIH. To the best of our knowledge, prior studies have not directly compared US- and MRI-measured ONSD in the same cohort of IIH patients.

## Materials and Methods

The research protocol followed the tenets of the Declaration of Helsinki and was approved by the Mayo Clinic Institutional Review Board. All patients were recruited and orally consented prospectively from the Neuro-Ophthalmology Service at Mayo Clinic, Rochester, MN, USA, from 1/1/2015 through 12/31/2016. Two groups of patients were identified based on funduscopic and clinical findings: pseudopapilledema (*n* = 22) versus papilledema from IIH (*n* = 22). In the pseudopapilledema group, 9 patients had optic disk drusen and 13 had small, cupless nerves as an etiology of the pseudopapilledema. The patients in the IIH group met diagnostic criteria based upon the revised criteria for IIH (32).

Exclusion criteria included: age <18 years, history of prior optic neuropathy, presence of CNS mass or other primary causes of increased ICP, dense media opacity, treatment with topiramate or acetazolamide for longer than 2 weeks before testing could be obtained, and failure to fulfill the revised diagnostic criteria for IIH (32). Two patients were included in the papilledema group who had been treated with acetazolamide for less than 2 weeks.

The patients in the papilledema group all underwent a similar battery of tests including a complete eye examination, brain MRI, lumbar puncture with opening pressure measurement done in the lateral decubitus position, OCT retinal nerve fiber layer imaging, B-scan US, and fundus/disk photos and autofluorescence imaging. All imaging was performed before the lumbar puncture, and the US ONSD was performed within 1 month of the MRI without any interim interventions. Only five of the patients in the pseudopapilledema group had a lumbar puncture, as it was otherwise not clinically warranted based on the presenting symptoms and exam findings in the remainder of the patients in the pseudopapilledema group. Similarly, some of the patients with optic disk drusen did not undergo MRI because the drusen were evident on neuro-ophthalmologic evaluation, and there were no concerning symptoms of raised ICP.

**Figure 1:**
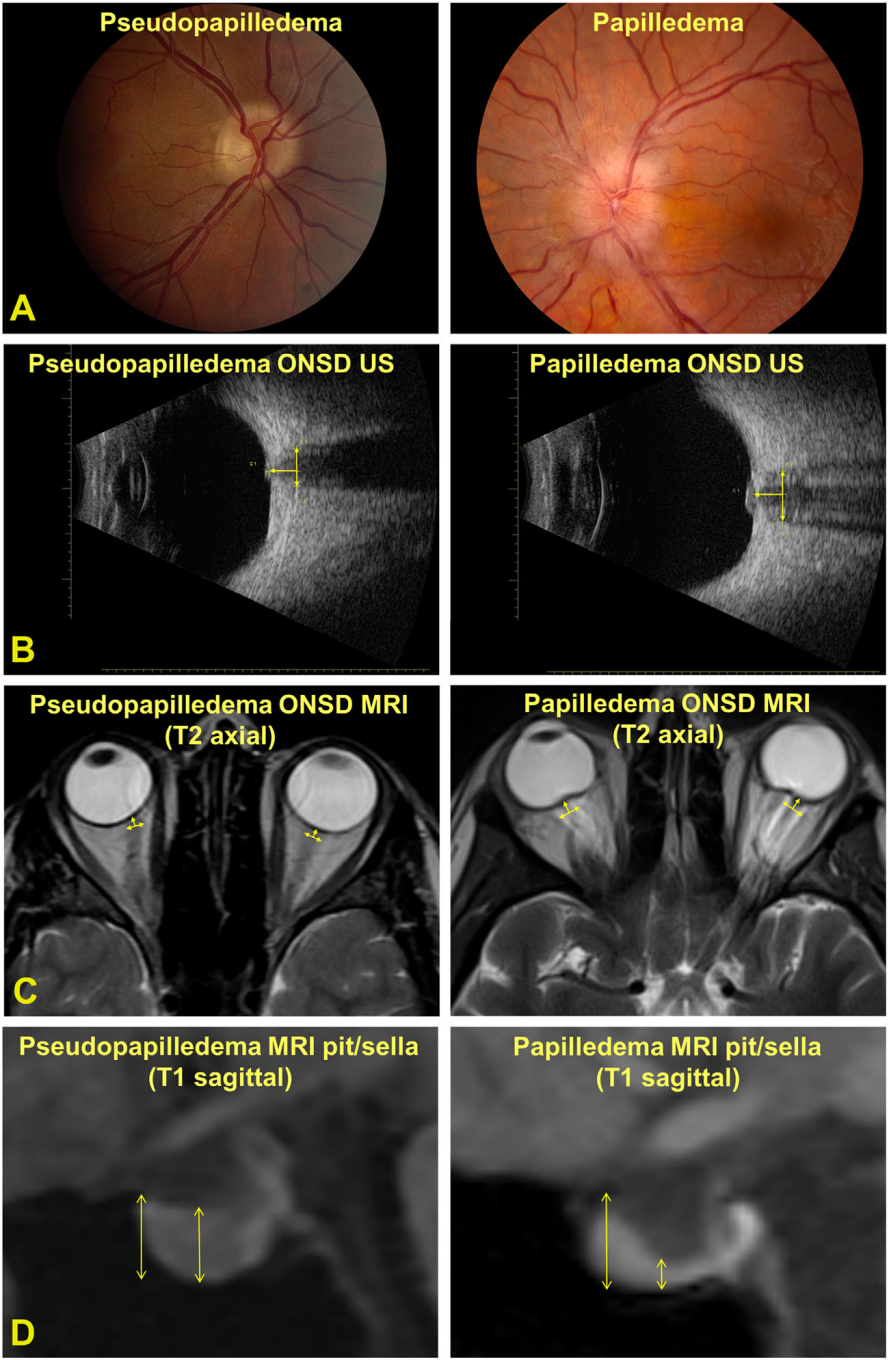
**(A)** Photographs of an optic nerve with pseudopapilledema (left panel) and papilledema (right panel). **(B)** ultrasonography- (US) measured optic nerve sheath diameter (ONSD) measured 3 mm posterior to the inner scleral surface. **(C)** Magnetic resonance imaging (MRI)-measured ONSD 3 mm posterior to the inner scleral surface. **(D)** MRI-measured pituitary (pit) and sella turcica (sella) measurements.

A single, masked technician with >30 years of experience with ocular ultrasonography performed all of the B-scan ultrasounds to avoid intra-tester variability. A coupling gel was used between the ultrasound probe (Quantel ultrasound, 10 MHz probe, axial resolution = 150 μm, lateral resolution = 300 μm) and the closed upper eyelid. The ONSD was measured 3 mm posterior to the inner scleral surface, measuring from outer wall to outer wall (Figure 1). The right and left optic nerves were measured in the horizontal, vertical, and oblique orientations yielding three measurements for each nerve, which were then averaged to obtain one value per patient. The patient was positioned supine and was instructed to look straight ahead for the horizontal and vertical ONSD measurements and, for the oblique ONSD measurement, was instructed to look toward 10:30 for the right eye and 1:30 for the left eye.

A board-certified neuroradiologist, with expertise in head and neck imaging, measured bilateral ONSD, pituitary, and sella turcica measurements on each MRI scan (not masked to the diagnosis). Scans were performed on various imaging systems (Siemens Healthcare, Erlangen, Germany; GE Healthcare, Boston, MA, USA; 1.5 and 3 T magnets) according to the general brain protocol, with high-resolution orbital images unavailable in the majority of cases. Therefore, MRI ONSD was measured using standard whole-brain axial T2-weighted images with in-plane resolution of 1–2 mm and slice thickness of 4–5 mm. MRI ONSD was defined for each eye 3 mm posterior to the inner sclera, measuring from outer wall to outer wall of the optic nerve sheath and averaged for each patient. In some cases, this required measuring on the axial slice above or below the image best demonstrating the sclera to obtain the most accurate measurement. Coronal planes were unavailable for the majority of imaging studies. On standard whole-brain sagittal T1-weighted images, the pituitary measurement was defined as the maximum vertical measurement in the middle 1/3 of the gland. The sella measurement was defined as the maximum vertical measurement of the entire sella turcica from the top of tuberculum sella to the sellar floor. The pit/sella ratio was calculated from each pair of measurements (Figure 1).

Statistical analysis was carried out using SAS software and R software [receiver-operator curve (ROC) analysis]. The optimal thresholds for detecting increased ICP for each parameter (US ONSD, MRI ONSD, and MRI pit/sella) were derived from ROC analysis. Sensitivity and specificity calculations were made using any combination of the three measured parameters to identify the most favorable combination of parameters for detecting increased ICP. Overall comparisons between the groups were completed with chi-square tests for categorical variables, while continuous variables were compared with two-sample *t*-tests. Overall correlations between parameters were calculated using Pearson correlation coefficients.

## Results

The pseudopapilledema group was 68% female compared with 86% female in the papilledema group, which was not a significant difference (Table 1). The average age was 41 years in the pseudopapilledema group compared with 32 years in the papilledema group, which also was not a significant difference (Table 1). The CSF opening pressure was significantly different (*p* < 0.001) for the pseudopapilledema group with an average of 14.5 (SD ± 2.8) cm H_2_O (*n* = 5) compared with 34.6 ± 7.7 cm H_2_O (*n* = 22) for the papilledema group (Table 1). The ONSD as measured by US and MRI, respectively, was 4.4 ± 0.7 and 5.2 ± 1.4 mm for the pseudopapilledema group compared with 5.2 ± 0.6 and 7.2 ± 1.6 mm for the papilledema group (*p* < 0.001 for both US and MRI, Table 1). The inter-eye difference for US-measured ONSD and MRI-measured ONSD was not statistically significant (Table 1). The average SD of the six US ONSD measurements in the whole group (*n* = 44) was 0.35 with a coefficient of variation of 7.6%, while the average SD of the two MRI ONSD measurements in the whole group (*n* = 40) was 0.27 with a coefficient of variation of 4.5%. The average MRI-measured pit/sella ratio was 0.7 ± 0.3 for the pseudopapilledema group compared with 0.3 ± 0.2 for the papilledema group (*p* < 0.001; Table 1).

**Table 1:** Comparison of selected parameters for patients with pseudopapilledema and those with papilledema from idiopathic intracranial hypertension.

Selected parameters	Pseudopapilledema (*n* = 22)	Papilledema (*n* = 22)	*p* -Value
Gender			0.15
Female	15 (68%)	19 (86%)	
Male	7 (32%)	3 (14%)	
Age (years)	41 ± 18	32 ± 10	0.1
Opening pressure (cm H_2_O)	14.5 ± 2.8^a^	34.6 ± 7.7	<0.001
Average US optic nerve sheath diameter (ONSD) (mm)	4.4 ± 0.7	5.2 ± 0.6	<0.001
Inter-eye difference	0.3 ± 0.2	0.3 ± 0.2	0.94
Average magnetic resonance imaging (MRI) ONSD (mm)	5.2 ± 1.4^b^	7.2 ± 1.6	<0.001
Inter-eye difference	0.3 ± 0.3^b^	0.4 ± 0.3	0.72
Average MRI pit/sella ratio	0.7 ± 0.3^b^	0.3 ± 0.2	<0.001

*^a^Value based on n = 5*.

*^b^Value based on n = 18*.

**Figure 2:**
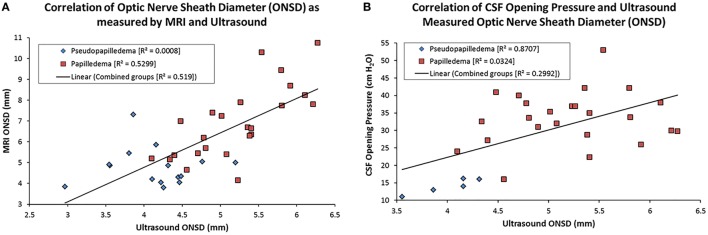
Scatter plots showing the correlation between magnetic resonance imaging (MRI)- and US-measured optic nerve sheath diameter (ONSD) **(A)** and cerebrospinal fluid (CSF) opening pressure and US ONSD **(B)**. The linear regression line on each is based on the combination of the papilledema and pseudopapilledema groups.

Magnetic resonance imaging- and US-measured ONSD yielded a strong positive correlation of 0.71 (*p* < 0.001, Table 2; Figure 2A). US and MRI ONSD, respectively, yielded moderate negative −0.40 and −0.49 correlations with the MRI pit/sella ratio (*p* = 0.01, *p* = 0.001, Table 2). Opening CSF pressure was moderately positively correlated with US and MRI ONSD, respectively, at 0.44 and 0.43 (*p* = 0.02, Table 2; Figure 2B), and a strong negative correlation of −0.57 was calculated between opening pressure and MRI pit/sella ratio (*p* = 0.002, Table 2).

**Table 2:** Correlations between selected parameters.

Selected parameters	*R*	*p* -Value
Opening pressure and US ONSD	0.44	0.02
Opening pressure and MRI ONSD	0.43	0.02
Opening pressure and MRI pit/sella	−0.57	0.002
MRI ONSD and US ONSD	0.71	<0.001
USONSD and MRI pit/sella	−0.40	0.01
MRI ONSD and MRI pit/sella	−0.49	0.001

*Correlations between selected parameters including optic nerve sheath diameter (ONSD) as measured by ultrasonography (US) or magnetic resonance imaging (MRI), MRI-measured pituitary-to-sella turcica ratio (pit/sella), and opening pressure of cerebrospinal fluid as measured during lumbar puncture*.

Using ROC analysis, we established optimal thresholds for establishing the likelihood of increased ICP. These thresholds include US-measured ONSD > 4.8 mm, MRI-measured ONSD > 6.0 mm, and MRI pit/sella ratio < 0.5. Sensitivity and specificity calculations were carried out for each individual measurement, as well as for every combination of imaging modalities, using the established threshold values (Table 3). US-measured ONSD > 4.8 mm yielded a sensitivity and specificity of 77 and 82%, respectively, while MRI-measured ONSD > 6.0 mm yielded a sensitivity and specificity of 77 and 83%, respectively (Table 3). A pit/sella ratio of <0.5, as a correlate of the “empty sella” sign, yielded a sensitivity and specificity of 82 and 67%, respectively (Table 3).

**Table 3:** Sensitivity and specificity based on combined modality imaging using established thresholds.

Selected parameters and combinations [#]	Sensitivity (%)	Specificity (%)	Area under the curve (AUC)
US ONSD	77	82	0.86
Threshold > 4.8 mm			
MRI ONSD	77	83	0.84
Threshold > 6.0 mm			
MRI pit/sella	82	67	0.90
Threshold < 0.5			
[1] US ONSD and MRI pit/sella	64	96	
[2] MRI ONSD and MRI pit/sella	68	96	
[3] MRI ONSD or MRI pit/sella	91	64	
[4] US ONSD or MRI ONSD or MRI pit/sella	96	50	
[5] US ONSD and MRI ONSD and MRI pit/sella	59	96	
[6] (MRI ONSD or US ONSD) and MRI pit/sella	73	96	

*Sensitivity and specificity calculations include ultrasonography- (US) or magnetic resonance imaging (MRI)-measured optic nerve sheath diameter (ONSD) and MRI-measured pituitary-to-sella turcica ratio (pit/sella). Threshold and AUC values are listed for detecting increased intracranial pressure in a patient with idiopathic intracranial hypertension*.

In combining the different measurement parameters, for the MRI parameters alone, namely, having either a threshold-level MRI ONSD or a threshold-level MRI pit/sella, the sensitivity was 91%, and the specificity was 64% (Table 3). Having a threshold-level value for any one of the three parameters alone yielded the highest sensitivity of 96% but lowest specificity of 50% (Table 3). Having a threshold-level US or MRI ONSD combined with a threshold-level MRI pit/sella yielded the highest combination of sensitivity and specificity at 73 and 96%, respectively (Table 3).

## Discussion

The results of this study show that MRI and B-scan US can both be used, either independently or in conjunction with one another, to establish the likelihood of increased ICP in patients with papilledema from IIH. To the best of our knowledge, this is the first study to date to compare both MRI- and US-measured ONSD in the same cohort of IIH patients.

Many others have explored the use of US to measure the ONSD as a correlate of increased ICP from various different etiologies (6–9, 11–14, 17–19, 21). The threshold ONSD for detecting increased ICP has ranged from 3.0 to 5.9 mm in previous studies (7–9, 11–13, 15, 16, 19, 33–36), which is in line with our established US ONSD threshold of >4.8 mm. Sensitivity and specificity values for increased ICP based on individual ONSD threshold levels have been calculated to range from 74.1 to 100% and from 38 to 99%, respectively (7–9, 11–13, 19, 21, 33–36).

This wide range in sensitivity and specificity values, along with differing propositions for the best ONSD threshold on US, is likely multifactorial. Part of this discrepancy may be due to these studies analyzing different patient populations, some with acutely raised ICP in the trauma setting, while only a few studied IIH, where the ICP likely increased more gradually. More importantly, ultrasonography is user- and technique-dependent, with different operators and probes producing variable image quality and resultant measurements. Many of these studies come from emergency medicine or critical care fields where a dedicated ultrasonographer is unavailable. Our sensitivity and specificity values for B-scan US-derived ONSD for detecting increased ICP in IIH patients compare favorably with the ranges found in the literature.

With respect to the MRI-measured ONSD, we found a sensitivity of 77% and specificity of 83% for ONSD at a threshold of 6.0 mm for detecting increased ICP. In comparison, Geeraerts et al. in 2008 calculated a sensitivity of 90% and specificity of 91% for detecting increased ICP > 20 mmHg using a threshold ONSD of 5.82 mm in patients with severe traumatic brain injury (26). In a cohort of IIH patients, Hoffmann et al. report a threshold range of ONSD, as measured at maximum width, from 5.5 to 5.6 mm, which yielded a sensitivity range of 72–80% and specificity of 95% for detecting increased ICP. The mean ONSD, again measured at maximal width, was reported to range from 5.88 to 6.09 mm in the same cohort of IIH patients (25). Our data are comparable to these studies with respect to threshold ONSD and sensitivity, although we obtained a slightly lower specificity value. Differences among studies may be related to technical differences between MRI scanners and protocols, location of measurements, and also the etiology and acuteness of the increased ICP.

There was a difference in absolute value of ONSD as measured by US and MRI, with a trend toward MRI-measured ONSD having a higher average compared with that measured on US (Table 1), despite being strongly correlated with each other (Table 2; Figure 2A). Reasons for this discrepancy likely relate to the difficulty in measuring the ONSD in precisely the same manner using different imaging modalities. US measurements of the optic nerve were taken in real time as an average of three planes in true perpendicular direction to the optic nerve sheath. The MRI examinations did not include isotropic high-resolution imaging of the orbits but rather whole-brain axial T2 images of lower resolution. On such images, the various layers of the optic nerve sheath cannot be resolved, and measurements were made based on the outer fibrous layer at its junction with periorbital fat. Finally, single measurements in the axial plane can vary with ocular position, reflecting physiologic tortuosity and slight obliquity relative to the true course of the nerve. Although the absolute values of ONSD differed between US and MRI, they provided almost identical sensitivities and specificities for detecting raised ICP.

In our assessment of the “empty sella” sign, which we determined by measuring the ratio of pituitary gland to sella turcica height, we report a sensitivity of 82% and specificity of 67% at a cutoff ratio of <0.5. Other studies have employed similar techniques for measuring the pituitary gland. Brodsky and Vaphiades report a sensitivity of 70% and specificity of 94% for the empty sella sign in diagnosing increased ICP in IIH. In their study, the empty sella was defined as the pituitary gland occupying <50% of the sella volume as well as a superior concavity configuration of the pituitary gland (28). Maralani et al. used the same definition and found this to have a sensitivity and specificity of 65.1 and 95.3%, respectively. Hoffmann et al. utilized a cutoff value of 4.8 mm for the pituitary gland height alone, which yielded a sensitivity and specificity of 88.0 and 69.57%, respectively (25). Finally, Yuh et al. report a sensitivity of 80% and specificity of 92% for moderate concavity of the pituitary, defined as the pituitary gland being between 1/3 and 2/3 the height of the sella, and also calculated a mean of 0.443 for the pituitary/sella height in the IIH group (31). Of note, the empty sella can be seen as a normal variant, particularly in patients with endocrine dysfunction or advanced age with global cerebral atrophy. Brodsky and Vaphiades found that the empty sella sign was seen in 5% of control patients (28), while Terano et al. noted that 19% of elderly patients displayed evidence of the empty sella sign (37). Therefore, it is important to utilize this ratio as an accessory measurement to ONSD to support the diagnosis of IIH in clinically suspected cases.

The best combination of sensitivity and specificity was obtained by having either a dilated US or MRI ONSD in combination with a sub-threshold MRI pit/sella ratio (Table 3, line [6]), which yielded a sensitivity of 73% and a specificity of 96%. An abnormality in any of the measurements provided a 96% sensitivity but only 50% specificity. Overall, these numbers may help guide the suspicion of raised ICP, but ultimately clinical examination and LP remain integral for the diagnosis of IIH. These US and MRI parameters may be helpful in detecting relapses of increased ICP, thus, potentially negating the need for repeat lumbar punctures after the initial diagnosis has been confirmed. Further studies are needed to assess the rapidity and degree of changes in the US and MRI parameters in response to changes in ICP in a cohort of IIH patients.

As the study cohort for the IIH group met all of the revised criteria for IIH (32), it is unclear how the results would apply to difficult or borderline cases of IIH in which the diagnosis may be uncertain. An additional limitation of the study is that the parameter threshold levels were derived from a relatively small cohort of IIH patients, thus making it difficult to apply those thresholds to the population as a whole.

US-measured ONSD has the advantage of being readily available in the clinic and ER settings as a rapid and focused screening examination of the orbits, which can identify important features such as enlargement of the ONSD and presence of optic disk drusen. However, it is important to note that our US ONSD data are based on measurements from an experienced echographer with 30+ years of experience who was blinded to the diagnosis. Many institutions do not have the luxury of having a dedicated echographer and, thus, the generalizability of our data may not be applicable to other practices. MRI enables evaluation of additional intracranial features of raised ICP, including the pit/sella ratio, effacement of subarachnoid space, transverse venous sinus stenosis, and detection of intracranial mass lesions. High-resolution MR of the orbits is also available when requested and provides information analogous to B-scan US, including optic nerve sheath tortuosity and flattening of the posterior sclera.

In conclusion, B-scan US provides an effective method of ONSD measurement and is a sensitive marker for increased ICP. In cases where the diagnosis is unclear, MRI can be ordered for detection of intracranial features of IIH, such as the empty sella sign, which can increase the specificity of diagnosis when combined with the ONSD measurements. Neither MRI nor US are appropriate replacements for lumbar puncture; rather, these tests serve as adjunctive tests that may help to confirm or refute the diagnosis of IIH in clinically ambiguous cases.

## Ethics Statement

The research protocol followed the tenets of the Declaration of Helsinki and was approved by an internal institutional review board.

## Author Contributions

DP and JC: designed and carried out study; interpreted and analyzed data; and wrote and edited/revised paper. M-LH: designed and carried out study; performed MRI imaging analysis and measurements; interpreted and analyzed data; and wrote and edited/revised paper. JL, NS, SH, EW, and DH: interpreted and analyzed data and edited/revised paper.

## Conflict of Interest Statement

The authors declare that the research was conducted in the absence of any commercial or financial relationships that could be construed as a potential conflict of interest.
